# Information Entropy in Chimera States of Human Dynamics

**DOI:** 10.3390/e27020098

**Published:** 2025-01-21

**Authors:** Franco Orsucci, Giovanna Zimatore

**Affiliations:** 1 Centre for Excellence in Mental Health Sciences, University of Amsterdam, 1105 AZ Amsterdam, The Netherlands; 2 Research & Development, Norfolk and Suffolk NHS Foundation Trust, Norwich NR6 5BE, UK; 3 Department of Theoretical and Applied Sciences, eCampus University, 22060 Novedrate, Italy; giovanna.zimatore@uniecampus.it

**Keywords:** information, pattern, coordination, synchronization, biosemiotics, free energy, chimera states, determinism

## Abstract

In human dynamics, functioning relies on intricate coordination patterns. Networks of synchronized oscillators in various biological and semiotic fields shape these dynamics. We have observed stability, instability, and transitions at multiple levels, indicating that coordination happens on all scales. We have examined coordination models for simplified and complex dynamics. In empirical research, we can frequently observe chimera states as the coexistence of coherence and incoherence, even in homogeneous networks. They are more evident in the heterogenous networks’ standard in human dynamics, where oscillators and nodes are mixed as different types. This paper proposes a simplified and overarching model for mixed chimeras. We discuss the information dynamics in these types of networks and their pattern transitions.

## 1. Introduction: Dynamic Patterns in Morphogenesis

The functioning of complex human systems relies on intricate coordination dynamics. Networks organized in biological and semiotic sub-systems shape these dynamics. Semiotics studies signs, symbols, and meaning-making processes, which are necessarily embodied and integrated with human biology. We have observed stability, instability, and changes at multiple levels, indicating that coordination happens on all scales [1]. Human complex systems show various patterns, interweaving multiple scales, from molecular biology to subjective and social dynamics [2]. Unique patterns emerge, change, and fill gaps through coupling and synchronization. In the networks, these patterns emerge and disappear, being restored or reconstructed in comparable or different ways. Synchronization and pattern formation dynamically organize complex systems. Synchronization represents time-organized activities, i.e., time patterns coevolving with spatial patterns. Space patterns and boundary morphology can facilitate different forms of synchronization. Information flows, spreads, or binds saturate new patterns to be released when pattern transitions occur [3]. Internal and external morphologies in animals and humans are necessarily connected to synchronization dynamics. For instance, the neural network architecture facilitates the rhythmic firing of neurons in the brain. Conversely, synchronization can also be a driver of pattern formation, as synchronized activity can lead to the emergence of new patterns [4]. The coordinated movement of cells during development can contribute to the formation of complex structures, such as the synchronization of neural networks. Neurons can fire synchronously, forming oscillatory patterns underlying cognitive functions, such as memory and perception. The connectivity patterns within neural networks influence emerging functional patterns [5], as the heart muscle cells must contract in a coordinated manner to pump blood effectively. Understanding pattern formation, transformation, and destruction in phase transitions is essential in various fields, including materials science, condensed matter physics, chemistry, ecology, biophysics, neuroscience, and biosemiotics. At the same time, patterns in individual subjects involve embodied complex multidimensional networks [6]. Complex patterns are mixed in human dynamics, as language is involved in scaling synchronization dynamics [7,8]. Language contributes to scaling synchronization dynamics by serving as both a medium for synchronization (e.g., coordinated speech) and a tool for regulating and amplifying synchronization across different levels of communication, from individual neural processes to social interactions. Mixed chimeras refer to states in dynamical systems where coherence and incoherence coexist across a network of oscillators, but with added heterogeneity. In such systems, the heterogeneity of oscillators is observed when the oscillators have different intrinsic properties (e.g., frequencies, coupling strengths, or node types), distinguishing them from homogeneous systems, where oscillators are identical. The coexistence of states is observed when certain regions or subgroups of the network exhibit synchronized (coherent) behavior, while others remain desynchronized (incoherent). These mixed chimera states are particularly relevant in human dynamics, as they mirror real-world systems, where interactions between diverse components (e.g., neurons, muscles, or social agents) produce different blends of order and disorder. In the results section, information dynamics will be introduced. Information dynamics refers to the study of how information is generated, transferred, stored, and transformed within a system over time. In human dynamics, information dynamics helps explain how coordination patterns emerge, stabilize, or transition, influenced by internal and external interactions.

## 2. Materials and Methods: Synchronization Dynamics in Human Pattern Formation

Scott Kelso proposed that unified coordination dynamics could be seen as a partnership between small, well-known synchronization models and large-scale synchronization models. The former is based on concepts in synergetics and nonlinear dynamics, such as the extended Haken–Kelso–Bunz (HKB) equation [4,9], and the latter on the statistical mechanics in ensembles of many oscillators [10]. Most research supporting the extended HKB model has involved coordinating only two interacting components, such as two joints of a single limb or two people interacting.

(1) Φ=δω−asin⁡ϕ−2bsin⁡2ϕ

In interaction dynamics, Φ is the phase or anti-phase synchronization in a dyadic interaction.

In contrast, Kuramoto captured the statistical mechanics features of large-scale coordination among many oscillators. Kuramoto’s model, in the hands of mathematical biologists such as Art Winfree [11] and Steven Strogatz [12], soon became a paradigm for large-scale coordination in complex living systems, which ranged from the flashing of fireflies to heart cells and neurons and concert audiences composed of human beings. This modeling and empirical work suggested that integrated patterns sustain a dynamic structure of synchronization and coordination, multistability, and metastability. The most popular form of the model has the following governing equation:

(2) $$\frac{d\theta_{i}}{dt}=\omega_{i}+\frac{K}{N}\sum_{j=1}^{N}sin\left(\theta_{j}-\theta_{i}\right),\;\;\;\;\;i=1\ldots .\;N,$$

The system comprises *N* limit-cycle oscillators with phase *θ_i_*, and coupling *K* describes their coupling strength (how strongly they interact with each other). $\omega_{i}$ is the natural frequency of the *i*-th oscillators at time *t.* Over time, various extensions and generalizations of the original Kuramoto model have been developed to account for more complex dynamics, such as oscillators that may have different coupling strengths and other oscillators (non-uniform coupling), or those that have different intrinsic frequencies and coupling strengths (non-identical oscillators). The effects of noise or random fluctuations can also be included to model real-world phenomena more accurately.

Later, Kuramoto and Battogtokh [13] observed that their previous model was based on ideal homogeneous forms of synchronization in large ensembles. In empirical research, we can frequently observe the coexistence of coherence and incoherence, even in a network of identical, complex, and non-locally coupled Ginzburg–Landau oscillators. Coupled non-identical oscillators were already known to exhibit mixed complex behavior (e.g., frequency locking, phase synchronization, partial synchronization, and incoherence). Identical oscillators were supposed to either synchronize in phase or incoherently drift. They showed that oscillators that were identically coupled with similar natural frequencies could behave differently from one another for specific initial conditions. Some could synchronize, while others remained incoherent in a stable state.

(3) $$\frac{\delta }{\delta t}\psi \left(x,t\right)=\omega \left(x\right)-\int G\left(x-x^{\prime }\right)\sin\left(\psi \left(x,t\right)-\psi \left(x^{\prime },t\right)+\alpha \right)dx^{\prime }$$

where *ω*(*x*) = *ω* for all *x*. Note that if *G*(*x*,*x*’) = exp (−|*x* − *x*’|), the kernel decays exponentially with the distance between *x* and *x*’, defining stronger coupling for nearby oscillators.

Abrams and Strogatz [14] named this mixed synchronization a chimera state, from the mythological Greek creature, made up of parts of different animals, and introduced some theoretical clarifications for such behavior. Chimera states are a fascinating phenomenon, emerging in complex coupled oscillator systems, thanks to the interplay between local and global coupling or other non-uniform structures. The extended Kuramoto model provides a mathematical framework to study them, revealing the subtle interactions that lead to the coexistence of synchronization and chaos. Chimera states were later found in limit-cycle oscillators, chaotic oscillators, chaotic maps, and neuronal systems. In the beginning, chimera patterns were observed in nonlocally coupled networks, but afterward, these states were also found in globally and locally (nearest neighbor) coupled networks and in modular networks. Additionally, the application of Markov chains to mapping couplings and chimera states was investigated. C.R. Laing studied chimera states in heterogeneous networks, analyzing the influence of heterogeneous coupling strengths. Of further interest for human dynamics is the emergence of chimera states in multiscale networks, which results from coupling different networks [15], as is usual in biosemiotic dynamics. The dynamic self-patterns’ hyper-structures result from networks of synchronized oscillators coupled in fields spanning heterogeneous biosemiotic, biological, and semiotic domains [16,17]. The ubiquity of the chimera mapping of synchronization and its different typologies has extended its original definition to areas that might include human nonidentical coupling oscillators in mixed heterogeneous networks and the multiscale networking-of-networks, which was already known to present chimera-like dynamics before this definition started to be used [8]. The dynamical integration of patterns includes fast and slow synchronization dynamics. For example, there will be fast physiological reactions in emotions, movements, neuro mediators, breathing, and heart rates, and slow reactions in neurotrophic factors, hormones, and attachment dynamics. Speech and cognition might be fast, moderate, or slow and, ideally, placed in a mesoscopic dynamic area [18]. Topological methods may provide additional insights into system coordination pattern dynamics that are irreducible to the properties of individual parts [7].

Recurrence plots are an effective technique for determining the degree of determinism, state transitions, and the complexity of systems [19,20]. Recurrence quantification analysis (RQA) is a nonlinear data analysis method that quantifies the number and duration of recurrences in a dynamical system based on its phase space trajectory. RQA can measure recurrent patterns, dynamics, and state transitions [21]. Santos et al. [22] demonstrated the diagnostic significance of RQA for chimera states. Laminarity, determinism, and recurrence rate demonstrate the distinction between chimera states and incoherent states. Recurrence plots effectively identify chimera states by visualizing the coexistence of coherent and incoherent dynamics within the system. Furthermore, changes in the recurrence patterns over time can be monitored to detect the collapse of chimera states, which can be identified by transitions from mixed patterns to uniform recurrence [22,23].

## 3. Results: Information Dynamics in Chimera Networks

Information dynamics in complex systems refers to how information flows, is processed, and evolves within systems composed of multiple interacting components. This field combines concepts from information theory, complex systems science, and dynamical systems theory. Key aspects include information transfer, storage, processing, and measurements in different contexts [24]. Chimera states are fascinating, complex systems phenomena that exhibit synchronized and desynchronized regions, presenting specific information dynamics. Chimera states are the norm in human dynamics as they present complex heterogeneous, hybrid, or mixed systems. This includes internal interactions between organs or subsystems and external biosemiotic interactions [25,26]. The dynamic mapping of heterogeneous synchronization in the brain indicates similar dynamics involving different brain areas related to emotional, motor, and verbal interactions. With pertinent implications for cognitive function, cognitive processes always require a balance between segregated and integrated neural processing. Segregation enables efficient computations in specialized brain regions, while integrated systems ensure coordinated and robust performance. Focused states tend to involve shorter local connections, while integration largely relies on subcortical regions and cortical hubs with diverse connections to other brain regions [27]. Recognizing chimera dynamics patterns can help to clarify the hybrid complexity of synchronization in critical cognitive states, where a balance between integration and segregation is required for adaptive cognition and social interactions [8]. Brain chimera dynamics might also be related to different neuronal interactions mediated by diverse types of synapses, fast and slow connections, and neuromodulators in the nervous system. Various types of neural interactions are an additional factor in the emergence of chimera dynamical states in human hybrid synchronization as separate regions interact to perform neurocognitive tasks. Variable patterns of partial synchrony form mixed chimera states replicated in different intersubjective interactions. Mixed chimera states are dynamical patterns in networks of heterogeneous coupled oscillators, where synchronous (coherent) and asynchronous (incoherent) behaviors coexist in distinct network regions. Mixed chimera states differ from classical ones in that they can simultaneously exhibit multiple synchronization patterns rather than the simple coexistence of one synchronized and one desynchronized region [27].

Human complex systems exhibit a rich tapestry of patterns, which are weaved in scaling with individual patterns and ecosystems. These patterns can be observed at various scales, from individual, dyadic interactions to social phenomena. The relationships between individuals and society can be visualized as networks, revealing connectivity patterns, clustering, and scaling [28]. Self-organization, where overall order arises from local interactions between parts of an initially disordered system, is significant in generating spontaneous order in complex systems [29]. This process can be spontaneous when sufficient information or energy is available and does not need to be controlled by any external agent. It is often triggered by seemingly random fluctuations and is amplified by positive feedback. The resulting organization is wholly decentralized and distributed over all system components. As such, it is flexible, robust, and able to survive or self-repair after substantial perturbation. Entropy represents the organization of a complex system. The term ‘entropy’ is borrowed from physics, where entropy is a measure of disorder. A cloud has higher entropy than an ice cube since a cloud allows for many more ways to arrange water molecules than a cube’s crystalline structure does. Analogously, a random message has a high Shannon entropy, as there are many possibilities for information organization. In contrast, a message that obeys a strict pattern has low entropy. There are also formal similarities in how entropy is calculated in physics and information theory. In physics, the formula for entropy involves taking a logarithm of possible physical states. In information theory, it is the logarithm of possible event outcomes. Shannon’s entropy [30,31] can best capture discrete probability distribution in stationary systems, quantifying overall system randomness, and it is defined as follows:

H = −Σ p_i_ log(p_i_) (4)

where p_i_ is the probability of state i, measured in bits (log base 2) or nats (ln).

Kolmogorov–Sinai (KS) entropy [32,33] measures the information rate generated in dynamical systems. It can quantify the complexity of system trajectories and the long-term temporal evolution. The mathematical definition of Kolmogorov–Sinai (KS) entropy is complex but fundamental to understanding dynamical system complexity:

h = lim[n→∞] (1/n) ln(N(ε,n))(5)

where,

- h represents the Kolmogorov–Sinai entropy;
- n is the number of observations or time steps;
- N(ε,n) is the minimal number of ε-distinguishable histories of length n in the system;
- ε represents a small resolution or precision parameter.

Conceptually, this formula quantifies the rate of information generation in a dynamical system. It measures the system’s complexity, predictability, and the convergence or divergence of dynamical trajectories.

We might consider the general effects of chimera states on information entropy [34,35,36] as follows:

1. Increased entropy in desynchronized regions. The desynchronized regions of a chimera state exhibit chaotic or random-like behavior, leading to higher entropy levels. This increased entropy can be seen as a source of information, disruption, uncertainty, or potential for systemic innovation. Stochastic areas are not expected to show well-structured patterns.
2. Reduced entropy in synchronized regions. The synchronized regions, on the other hand, exhibit more ordered behavior, leading to lower entropy levels. This reduced entropy can be seen as a loss of information or a decrease in the system’s ability to adapt to changes. Predictability will be higher, and patterns will be more evident and stable.
3. Overall entropy. The overall entropy of a chimera state depends on the balance between the synchronized and desynchronized regions. If the desynchronized regions dominate, the overall entropy will be higher. If the synchronized regions dominate, the overall entropy will be lower. The coexistence of order and disorder in chimera states can enhance a system’s information processing capabilities and flexibility. The synchronized regions can provide a stable background for information transmission, while the desynchronized regions can introduce noise and variability, stimulating systemic creativity and innovation. Chimera states can also improve communication by balancing coherence and diversity. The synchronized regions can ensure reliable communication, while the desynchronized regions can introduce new options and systemic perspectives. The desynchronized regions can introduce noise and variability, improving the signal-to-noise ratio. The dynamic nature of chimera states allows the network to adapt to changing conditions. The network can adjust its information processing capabilities accordingly as the balance between synchronized and desynchronized regions shifts.

The systemic benefits of chimera states include increased system resilience and robustness, improved fault tolerance, and enhanced adaptability [37,38]. The presence of both synchronized and desynchronized regions can make the network more resilient to disturbances and failures. If one part of the network is disrupted, the other parts can continue functioning, ensuring the overall system’s stability. Switching between synchronized and desynchronized states can help the network adapt to changes in the environment, such as variations in input signals or network topology.

The chimera states observed in biological systems, such as the brain, have inspired new computational paradigms [25,36]. By mimicking these dynamics in artificial networks, researchers can develop novel computing architectures that are more efficient and robust. While significant progress has been made in understanding the mechanisms underlying chimera states, further research is needed to develop effective strategies for facilitating their self-organization. Studying signs and meaning in living organisms in biosemiotics provides a fascinating lens through which to view chimera states. These complex dynamical patterns, with the coexistence of synchronized and desynchronized regions, can manifest the various forms of organization in biological systems. Chimera states can increase the information processing capacity of a biosemiotic network by allowing for both synchronous and asynchronous communication channels. The desynchronized regions can introduce noise, disrupt information transmission, and lead to innovative solutions and creative thinking. The dynamic nature of chimera states allows the network to adapt to changing environmental conditions and information demands.

Chimera states may have evolved to increase the complexity and adaptability of biological systems [39]. Genetic and epigenetic factors can influence the emergence and stability of chimera states in biological networks. In human societies, chimera states can influence the evolution of culture and language. Understanding the role of chimera states in brain function can lead to new insights into cognitive processes, consciousness, and neurological disorders [25]. In considering human pattern dynamics, we took a biosemiotic stance, overarching the different types of networks and oscillators involved. These oscillators can be continuous or discrete, and their coupling can be linear or nonlinear. The networks involved will be heterogeneous, and the coupling strength and function can vary among different pairs of oscillators. The system exhibits complex spatiotemporal patterns, with regions of synchronized and desynchronized activity. The network’s collective emergent behavior can significantly differ from individual oscillators. The underlying network topology, such as the degree distribution and clustering coefficient, can also play a crucial role in shaping the dynamics of mixed-node chimera networks. Noise and border conditions can further enrich the dynamics of mixed-node chimera networks.

In consideration of previous research [35,36,37], a simplified general mathematical model for a heterogenous mixed chimera network in human dynamics can be developed as follows:

(6) $$\frac{{\;dx}_{i}\;}{dt}=F_{i}\left(x_{i}\right)+\sum_{j}\;K_{ij}G_{ij}(x_{j,}x_{i})$$

where,

- *x_i_* is the state vector of the *i*-th oscillator;
- *F_i_*_(_*x_i_*_)_ is the intrinsic dynamics of the *i*-th oscillator, which can differ for different oscillators;
- *K_ij_* is the coupling strength between the *i*-th and *j*-th oscillators;
- *G_ij_*(*x_j_*, *x_i_*) is the coupling function between the *i*-th and *j*-th oscillators, which can differ for different oscillators.

This equation is a coupled differential equation—or an interaction-based differential equation. More specifically, it represents a generalized form of a coupled, nonlinear dynamical system. The general structure suggests a system where each component evolves due to its internal dynamics, while simultaneously being influenced by interactions with other components in the system. The coupling allows interactions among multiple variables. Variables are interconnected and influence each other, although, each variable has independent dynamics. The change rate depends on the variable’s intrinsic behavior and its interactions with different variables.

By studying the mathematical models of mixed-node chimera networks, we can gain deeper insights into the mechanisms underlying their emergence and stability. This knowledge can be applied to various fields, including neuroscience, biosemiotics, engineering, and physics. The ubiquity of the chimera mapping of synchronization and its different typologies has extended its original definition to areas that might include human nonidentical coupling oscillators in hybrid networks and the multiscale networking of networks that were already known to present chimera-like dynamics before this definition started to be used [8]. In essence, while hybrid chimera networks focus on the diversity of coupling within a single type of oscillator, heterogenous, mixed-node chimera networks explore the complex interplay between oscillators of different types. The dynamical integration of patterns will include fast and slow synchronization dynamics. For example, there will be fast physiological reactions in emotions, movement, neuro mediators, breathing, and heart rate, and slow reactions in neurotrophic factors, hormones, and attachment dynamics. Speech and cognition might be fast, moderate, or slow, and, ideally, will be placed in a mesoscopic dynamic area [18].

## 4. Discussion: Self-Organization and Pattern Dynamics

The dynamic mapping of heterogeneous synchronization in the brain indicates similar dynamics, involving different brain areas that are related to emotional, motor, and verbal interactions. With pertinent implications for cognitive function, cognitive processes always require a balance between segregated and integrated neural processing. Segregation enables efficient computations in specialized brain regions, while integrated systems ensure coordinated, robust performances. Focused states tend to involve shorter, local connections, while integration largely relies on subcortical regions and cortical hubs with diverse connections to other brain regions [25,26,27]. Recognizing chimera dynamics’ patterns can help to clarify the hybrid complexity of synchronization in relevant cognitive states, where a balance between integration and segregation is required for adaptive cognition and social interactions [8]. Brain chimera dynamics might also be related to different neuronal interactions, which are mediated by different types of synapses, fast and slow connections, and neuromodulators in the nervous system [39]. Various types of neural interactions are undoubtedly an additional factor in the emergence of chimera dynamical states in human synchronization. As separate regions interact to perform neurocognitive tasks, variable patterns of partial synchrony form chimera states. Pattern formation, transitions, and creative destruction and reconstruction form cycles of multiscale, couplings, and de-couplings [40]. These transitions test the systemic flexibility and resilience of the complex coordination of sub-systems. Phase transitions between patterns release free information entropy until a new organization coalesces into new patterns. The coupled inter-subjective organization tolerates the transient states of increased entropy associated with decoupling or pattern dissolution and transitions facilitating new morphogenesis. Uncertainty, dissonance, distress, or surprise might emerge with related risks, but they may also provide opportunities for new positive self-organization.

Recent work by Tschacher and Haken [41] offers a compelling perspective based on the Fokker–Planck equation also called the Kolmogorov forward equation.

(7) $$\frac{dP(x;t)}{dt}=\frac{d}{dx}\left(k\left(x-x_{0}\right)P\left(x;t\right)\right)+Q\frac{d^{2}P\left(x;t\right)}{dx^{2}}\;=\;D\;+\;S$$

The Fokker–Planck equation summarizes the following ideas: the temporal change in the probability of a variable *x* (i.e., the left-hand side of the equation) can be modeled by the sum of a deterministic term, *D*, and a stochastic term, *S*, contributing to such change. We see that the *S* term contains *d* to the square. This means we are dealing with the second derivative of probability *P*, i.e., with the diffusion (the variance) of *P*(*x*). *Q* is the parameter that models the increase or decrease in the variance of variable *x* over time. The *D* term correspondingly describes the first derivative, i.e., the change of the probability of a given value of *x*, i.e., *P*(*x*). In this respect, *k* and (x-x_0_) represent factors that directly modify *P*. If a system has a stable equilibrium state (i.e., the system has an attractor), *k* specifies the force by which the system state, *x*, is driven to this equilibrium state. *k* is the force that restores the equilibrium whenever the system has been pushed outside its attractor by a random fluctuation or by an intervening force or action from outside. The Fokker–Planck equation suggests that behavior change is commonly a mixture of stochastic and deterministic processes. Human relations are affected by deterministic (causation) and stochastic (chance) forces. Deterministic processes refer to defining the boundary conditions. Stochastic processes refer to a broader exploration of states and possibilities in the bipersonal field state space. Thanks to stochastic dynamics, a relational experience can escape being trapped in a particular range of disharmonic, perceptual, and affective experiences. Stochastic dynamics are related to the emergence of uncertainty, dissonance, distress, novelty, surprise, opportunities, and, sometimes, playful humor [42].

The free energy principle, formulated by neuroscientist Karl Friston, suggests that all living systems (including the human brain) aim to minimize a quantity known as “free energy”. This principle explains how organisms perceive and act in their environment to maintain stability and avoid uncertainty. It refers to information and thermodynamic entropy as applied to cognitive neuroscience. It is strictly related to synchronization and pattern formation [43,44]. The free energy principle suggests that living systems, such as the brain, aim to minimize their free energy and information entropy by optimizing their internal models of the environment. This optimization process can be formalized using the principles of predictive coding, which are closely related to Bayesian inference. The emergence of dynamic patterns in coordination can be understood within the free energy principle and Bayesian inference framework. This formulation suggests that perception inherently tends to induce dynamical instabilities, which enable the brain to respond sensitively to sensory perturbations. By minimizing free energy, the system self-organizes to maintain optimal coordination patterns for survival and adaptation. At the same time, self-organized arousal and metastability enable perceptual transitions in Bayes-optimal perception [45,46]. Bayes’ theorem provides a mathematical understanding of how the system can infer the hidden causes of the coordination patterns and update its internal model accordingly. Integrating Bayes’ theorem, the free energy principle, and the study of dynamic patterns has far-reaching implications across various disciplines, including neuroscience, machine learning, and complex systems theory. In cognitive neuroscience, this framework helps explain the brain’s ability to maintain and adapt coordination patterns in neural activity to optimize information processing and decision-making. In artificial intelligence and robotics, Bayesian models and the free energy principle can inform the design of more adaptive and energy-efficient control systems, which can self-organize and maintain optimal coordination patterns. By understanding the role of Bayes’ theorem in the context of the free energy principle and the emergence of dynamic patterns, we can gain a more comprehensive and coherent understanding of the fundamental mechanisms underlying the self-organization and adaptation of complex systems.

Pattern transitions are frequently related to metastability. This has been found in the esthetic experience of ambiguous forms [47], beliefs [48], or musical dissonance [49] in cognitive neuroscience. The Wundt Curve, named after Wilhelm Wundt, is a bell-shaped curve illustrating the relationship between stimulus intensity and its effect. The Wundt Curve explains the balance between predictability and complexity [50,51]. As a stimulus intensity (e.g., shapes, light, or sound), its effect can be sensory-neutral, pleasant, or unpleasant. The Wundt Curve represents an optimal informational balance between determinism and stochasticity, where physiological arousal reaches curiosity and pleasure, the pink noise spot, avoiding the opposite negatives of brown and white noise. The sweet spot between the two is usually governed by an inverted U-shaped curve, which peaks in the middle. The peak position may vary based on individual perceptions, but the pattern remains consistent for most perceivers [52,53]. Like the Wundt Curve, the Bayes theorem represents a balance between the order and disorder of pattern formation in human dynamics. The Bayes theorem provides a framework for updating one’s beliefs (posterior probabilities) based on new evidence (likelihood) and prior knowledge (prior probabilities). The Bayes theorem can be used in complex systems to construct probabilistic models, which describe the system’s dynamics and internal representations of the external world. As in the case of the Wundt Curve, we can consider that the Bayes theorem is embedded as a functioning information balance in human dynamics. By balancing the free energy (or prediction error), the complex system effectively performs Bayesian inference to improve its predictive capabilities and maintain an accurate internal representation of the external world.

## 5. Conclusions

The very nature of complex adaptive systems is that they must remain flexible and responsive to environmental changes over time. A system that becomes too rigidly optimized and reaches a very low entropy may become vulnerable to disruption or the “creative destruction” of its existing patterns. As a system approaches this critical state of low entropy and high coordination, it becomes increasingly rigid, fragile, and sensitive to external influences or internal fluctuations. These critical fluctuations can then trigger a phase transition, in which the system abandons its current optimal patterns and undergoes a period of creative destruction, exploring new organizational possibilities [54,55,56].

Complex systems often operate near a critical point or phase transition, where small perturbations can lead to large-scale reorganization of the system’s structures and dynamics. When the system’s current patterns become insufficient or inflexible, the free energy principle may drive it to explore new organizational possibilities, even temporarily increasing its entropy. This cycle of optimization, rigidity, and creative destruction can be seen as a fundamental feature of complex adaptive systems that strive to maintain their structural and functional integrity over time.

Chimera states demonstrate a unique form of resilience through their inherent structural flexibility. The coexistence of synchronized and desynchronized regions allows the system to maintain partial functionality, even when some components are disrupted. This partial synchronization acts as a dynamic buffer against complete system breakdown. The mixed synchronization pattern enables robust information transfer across different system components. Synchronized clusters can maintain coherent communication, while desynchronized regions provide adaptive pathways for alternative signal routing.

Unlike completely synchronized or random systems, chimera states occupy an intermediate complexity zone with dynamic flexibility. This intermediate state enhances resilience by allowing the localized stabilization of relevant system components. Due to their unique balance between determinism and stochasticity, chimera states result in distributed response mechanisms and graceful degradation under perturbation. The resilience of chimera states relies on their unique network architecture. Different coupling strengths, network connectivity patterns, and node characteristics significantly modulate the system’s ability to maintain coherence under systemic stress.

Chimera states can dynamically reconfigure their synchronization patterns in response to external perturbations. This adaptive capacity allows the system to redistribute energy, information, or computational resources in real-time. Chimeras exhibit a distinctive information transfer mechanism, where synchronized regions maintain coherent, predictable information flows, while desynchronized regions introduce probabilistic information pathways. This creates a multi-scale information processing architecture. The coexistence of ordered and disordered regions generates unique entropy characteristics. Synchronized clusters reduce local entropy, and desynchronized (decoupled) regions increase local entropy.

The result is a complex information landscape with mixed predictability. Synchronized regions follow predictable, rule-based interactions and demonstrate precise coupling mechanisms. Desynchronized regions introduce randomness, allow for adaptive, flexible system responses, and enable the exploration of alternative dynamical configurations. Synchronization-breaking mechanisms and parametric perturbations can produce pattern destruction processes. Noise introduction, coupling strength modifications, or topological network changes can trigger pattern destructions and creative transformations. These information entropy dynamics have interdisciplinary relevance in cognitive neuroscience, ecosystems, and biophysics. Related morphogenetic perspectives apply in patterns of embodied cognitive processes, structural coupling between organisms and environments, and information gradients driving developmental dynamics.

Chimeras represent a unique class of complex dynamical systems characterized by coexisting coherent (synchronized) and incoherent (desynchronized) regions. This hybrid or mixed state bridges deterministic and stochastic behaviors, offering a rich landscape for understanding information processing and system dynamics. Combining multidisciplinary expertise in human dynamics is crucial to making meaningful progress in this new frontier of dynamical systems studies.

## Data Availability

The data presented in this study are available on request from the corresponding author.
